# Interaction of Antifungal Drugs with CYP3A- and OATP1B-Mediated Venetoclax Elimination

**DOI:** 10.3390/pharmaceutics14040694

**Published:** 2022-03-23

**Authors:** Eric D. Eisenmann, Dominique A. Garrison, Zahra Talebi, Yan Jin, Josie A. Silvaroli, Jin-Gyu Kim, Alex Sparreboom, Michael R. Savona, Alice S. Mims, Sharyn D. Baker

**Affiliations:** 1 Division of Pharmaceutics and Pharmacology, College of Pharmacy, The Ohio State University, Columbus, OH 43210, USA; eisenmann.11@osu.edu (E.D.E.); garrison.220@buckeyemail.osu.edu (D.A.G.); talebi.9@osu.edu (Z.T.); jin.1134@osu.edu (Y.J.); silvaroli.1@buckeyemail.osu.edu (J.A.S.); kim.6182@buckeyemail.osu.edu (J.-G.K.); sparreboom.1@osu.edu (A.S.); 2 Vanderbilt-Ingram Cancer Center, Division of Hematology and Oncology, Vanderbilt University School of Medicine, Nashville, TN 37232, USA; michael.savona@vumc.org; 3 Division of Hematology, Department of Medicine, The Ohio State University, Columbus, OH 43210, USA; alice.mims@osumc.edu

**Keywords:** venetoclax, OATP1B1, antifungal, pharmacokinetics, CYP3A

## Abstract

Venetoclax, a BCL-2 inhibitor used to treat certain hematological cancers, exhibits low oral bioavailability and high interpatient pharmacokinetic variability. Venetoclax is commonly administered with prophylactic antifungal drugs that may result in drug interactions, of which the underlying mechanisms remain poorly understood. We hypothesized that antifungal drugs may increase venetoclax exposure through inhibition of both CYP3A-mediated metabolism and OATP1B-mediated transport. Pharmacokinetic studies were performed in wild-type mice and mice genetically engineered to lack all CYP3A isoforms, or OATP1B2 that received venetoclax alone or in combination with ketoconazole or micafungin. In mice lacking all CYP3A isoforms, venetoclax AUC was increased by 1.8-fold, and pretreatment with the antifungal ketoconazole further increased venetoclax exposure by 1.6-fold, despite the absence of CYP3A. Ensuing experiments demonstrated that the deficiency of OATP1B-type transporters is also associated with increases in venetoclax exposure, and that many antifungal drugs, including micafungin, posaconazole, and isavuconazole, are inhibitors of this transport mechanism both in vitro and in vivo. These studies have identified OATP1B-mediated transport as a previously unrecognized contributor to the elimination of venetoclax that is sensitive to inhibition by various clinically-relevant antifungal drugs. Additional consideration is warranted when venetoclax is administered together with agents that inhibit both CYP3A-mediated metabolism and OATP1B-mediated transport.

## 1. Introduction

Venetoclax (ABT-199, Venclexta), a first-in-class BCL-2 inhibitor, has transformed the treatment of hematologic cancers, including chronic lymphocytic leukemia, small lymphocytic lymphoma, and acute myeloid leukemia (AML) [1]. Despite impressive response rates with notable improvements in survival [2], treatment with venetoclax is limited by the occurrence of serious adverse events, including tumor lysis syndrome, neutropenia, and thrombocytopenia. The clinical utility of venetoclax is further complicated by a highly variable pharmacokinetic profile with nearly 15-fold variation in apparent oral clearance at approved doses, and a drug-drug interaction (DDI) liability that may predispose some patients to exacerbated adverse events [3].

The mechanisms underlying the unpredictable pharmacokinetic profile and DDI potential of venetoclax remain largely undefined. It has been speculated that a critical determinant of this pharmacokinetic variability is associated with differential expression of polymorphic drug-metabolizing enzymes (DME) and/or transporters at sites of elimination. The pathway mediating the hepatic metabolism of venetoclax has been reasonably well established and involves the DME subfamily CYP3A [4]. Based on these data, DDI between venetoclax and azole antifungals (e.g., ketoconazole [5], posaconazole [6]) are hypothesized to be primarily mediated by CYP3A inhibition. However, the mechanisms by which venetoclax is taken up into human liver cells are still largely unknown. Given that CYP3A inhibitors may inhibit drug transporters [7], the mechanisms underlying the hepatic uptake of venetoclax may be relevant to DDI. In the context of routine patient care, it should be emphasized that venetoclax is frequently administered together with other anticancer drugs [8] or with prophylactic antifungal drugs [9] that may impact the metabolism and/or transport of venetoclax and cause unintentional DDIs. Additionally, interpatient variability in the expression and function of DMEs and drug transporters likely contributes to the observed pharmacokinetic variability with venetoclax [10]. While the venetoclax dose is adjusted based on the potential for a concomitant drug to inhibit CYP3A, it remains possible that differential inhibition of hepatic uptake transporters is relevant to potential DDI with venetoclax. However, while the ability of older azole antifungals (e.g., ketoconazole) to inhibit hepatic uptake transporters has been established, the impact of newer, clinically-relevant antifungals (e.g., posaconazole, isavuconazole, micafungin) on hepatic uptake transporters has not been determined. Overall, a rational justification for the current venetoclax dosing recommendations is lacking, and mechanistic details of the exact sites of interaction remain poorly characterized.

The aim of the present study was to determine the relative contributions of DMEs and transporters to the pharmacokinetics of venetoclax in various genetically-engineered mouse models, and to characterize the mechanisms underlying the DDI potential of venetoclax with antifungals. By selectively removing DME or drug transporters, genetically engineered mice allow for precise determination of the mechanisms underlying drug disposition and DDI [11].

## 2. Materials and Methods

### 2.1. Chemical and Reagents

Venetoclax, rifampin, ritonavir, cobicistat, ketoconazole, fluconazole, itraconazole voriconazole, posaconazole, isavuconazole, micafungin, caspofungin, and anidulafungin were purchased from MedChemExpress (Monmouth Junction, NJ, USA). The major itraconazole metabolites, hydroxy-itraconazole and keto-itraconazole were kindly provided by Dr. Nina Isoherranen (University of Washington, Seattle, WA, USA). [^3^H]Estradiol-17β-D-glucuronide (EβG; specific activity, 50.1 Ci/mmol), a positive control substrate for OATP1B, was purchased from American Radiolabeled Chemicals (Saint Louis, MO, USA). [^3^H]Venetoclax (specific activity, 29.3 Ci/mmol) was obtained from ViTrax (Placentia, CA, USA). PEG400 was purchased from Sigma-Aldrich (Burlington, MA, USA). Dimethyl sulfoxide (DMSO), LC-MS-grade formic acid, methanol, and acetonitrile were purchased from Fisher Scientific (Fair Lawn, NJ, USA), and 8-acetoxy-trisulfopyrene was obtained from Carbosynth Limited (Compton, Berkshire, UK). Human recombinant CYP3A4 Supersomes (456207) was purchased from Corning (Glendale, AZ, USA). NADPH RapidStart Regenerating System was purchased from SEKISUI XenoTech (Kansas City, KS, USA).

### 2.2. In Vitro Metabolism Studies

The ability of human CYP3A4 to metabolize venetoclax was assessed using human CYP3A4 supersomes using standard procedures [12]. Reactions were performed in a volume of 100 µL. Reactions contained 40 nM supersomes, 10 µM venetoclax, 100 mM potassium phosphate buffer (pH 7.4), 3 mM MgCl_2_, and 10 µM ketoconazole or an equivalent volume of vehicle (DMSO). The mixture was preincubated for 5 min at 37 °C before initiating the reaction by the addition of the NADPH regenerating system and incubating at 37 °C with continuous shaking (400 rpm). At 0 h and 2 h, a 20 µL aliquot was mixed with 20 µL methanol to terminate the reaction. The mixture was then vortexed and stored at −80 °C until analysis. Venetoclax was quantified using a validated UHPLC-MS/MS analytical method [13].

### 2.3. In Vivo Pharmacokinetic Studies

In vivo experiments were performed with female and male mice from inbred wild-type strains (FVB, DBA) or with age- and sex-matched genetically-engineered mice with a deficiency of the Cyp3a locus (8-genes; FVB.129P2-*Cyp3a13^tm1Ahs^* Del(5Cyp3a57-Cyp3a59)1Ahs) [14] [CYP3A(−/−)], or the hepatic uptake transporter OATP1B2 [15] [OATP1B2(−/−)], the single murine orthologue of human OATP1B-type transporters. All mice used had a body weight of 20–30 g and were 10–20 weeks of age. The studies were conducted in accordance with the NIH’s Guide for the Care and Use of Laboratory Animals and as approved by the University Laboratory Animal Resources Animal Care and Use Committee at Ohio State University (2015A00000101-R2; last approved 20 September 2021) and. Mice were housed in a temperature- and light-controlled environment with free access to water and a standard diet, excluding a 3-h fast before venetoclax administration. For in vivo studies, venetoclax was prepared for oral administration by dissolving the powder in DMSO (5%) and then adding PEG300 (50%), Tween-80 (5%), and water (40%) stepwise to create a suspension with a final concentration of 2 mg/mL. Ketoconazole was prepared for oral administration by preparing a 10 mg/mL suspension in PEG400. Micafungin sodium was dissolved in PBS at a concentration of 20 mg/mL. Based on our previous experience, all experiments were prospectively designed to have group sizes of five; however, pharmacokinetic data from two CYP3A(−/−) mice were excluded due to tolerability concerns, resulting in a final group size of three (Supplementary Table S1).

Pharmacokinetic studies were performed as described previously [16]. In brief, approximately 30 µL of whole blood was collected from each mouse at six time points between 5 min and 8 h after venetoclax administration; in most cases, samples were collected at 5 min, 15 min, 30 min, 1 h, 3 h, and 8 h. The first three samples were collected from a submandibular vein using a sterile 5-mm Goldenrod Animal Lancet and a heparinized capillary tube. The fourth and fifth samples were collected from the retro-orbital venous plexus using capillary tubes after anesthetizing the mice using 2% isoflurane. The terminal sample was collected into a 1.3-mL tube containing heparin by cardiac puncture using a syringe and needle. Whole blood samples were centrifuged at 13,000× *g* for 5 min. The plasma supernatant was then collected, immediately placed on dry ice, and stored at −80 °C until analysis. Venetoclax was quantified using a validated UHPLC-MS/MS analytical method [13]; this method utilizes 5-µL sample volumes and has a lower limit of quantification of 5 ng/mL.

### 2.4. Pharmacokinetic Data Analysis

Non-compartmental analysis was performed to determine the pharmacokinetic parameters of venetoclax using Phoenix WinNonlin version 8.0 (Certara, Princeton, NJ, USA). Peak plasma concentration (C_max_) was determined by visual inspection of the data from the log concentration versus time curves. The linear trapezoidal rule was used to obtain the area under the plasma concentration-time curve from time zero to the time of the last measurable concentration (AUC_0-last_).

### 2.5. In Vitro Transport Assays

Transport assays assessing the uptake of venetoclax in the presence or absence of a panel of antifungal drugs and other known transport inhibitors were conducted as previously described [12]. In brief, radioactive uptake assays were performed utilizing HEK293 cell lines stably expressing OATP1B1 or OATP1B2, or cells expressing the respective vector control (VC) [17,18]. The generation of stable, isogenic Flp-in T-Rex293 cells expressing OATP1B1*1a (wild-type) has been described previously [18]. Cells were cultured in Dulbecco’s Modified Eagle Medium (DMEM; Invitrogen) supplemented with 10% FBS; the media for cells expressing OATP1B1 or VC was supplemented with hygromycin B (25 mg/mL; Invitrogen), and blasticidin (37.5 mg/mL; Biovision).

For experiments seeking to directly determine if venetoclax is an OATP1B1 substrate, cells overexpressing OATP1B1 or VC were seeded in six-well plates in phenol red-free DMEM containing 10% FBS and doxycycline (1 µg/mL) for 24 h. Cells were washed with warm PBS and incubated with 0.2 µM radiolabeled EβG, a probe OATP1B substrate, or 1 µM radiolabeled venetoclax in phenol red-free DMEM (without FBS and supplements) for 15 min. The experiment was terminated by washing three times with ice-cold PBS. Cells were then immediately lysed with 1 mL of 1 N NaOH for 4 h on an orbital shaker at room temperature to measure total radioactivity. Alternatively, cells were trypsinized with 200 µL of TrypLE (Thermo Fisher Scientific, Waltham, MA, USA) for 3–5 min, rinsed with 800 µL of ice-cold washing buffer, and ProteoExtract Native Membrane Protein Extraction Kits (EMD Millipore, Burlington, MA, USA) were used to separate membrane and intracellular fractions. Dissociated cells were then transferred to 1.5-mL Eppendorf tubes. For measurement of total radioactivity, cells were neutralized with 500 µL of 2 M HCl after 4 h. For measurement of total radioactivity in the membrane or intracellular fraction, cells were isolated following the manufacturer’s protocol, as we have reported previously [19]; this procedure is based on a differential extraction procedure under non-denaturing conditions. Radioactivity was measured by a liquid scintillation counting, and total protein was quantified using a Pierce BCA Protein Assay Kit (Thermo Fisher Scientific). Uptake was calculated by dividing the disintegrations per minute (dpm) from each replicate by the amount of protein (mg).

For inhibition experiments, cells were seeded in 12- or 24-well plates in phenol red-free media and were incubated at 37 °C for 24 h; cells overexpressing OATP1B1 or VC were incubated with doxycycline (1 µg/mL) for 24 h. Cells were washed with warm PBS and pre-incubated with the indicated concentrations of each potential inhibitor (or an equal volume of vehicle) in phenol red-free DMEM (without FBS and supplements) at 37 °C for 15 min. Cells were then incubated with phenol red-free DMEM containing the indicated concentration of each antifungal drug and 0.2 µM EβG for an additional 15 min. The experiment was terminated by washing three times with ice-cold PBS. Cells were lysed with 1 N NaOH overnight at 4 °C, and the solution was neutralized with 2 M HCl. As before, intracellular EβG concentrations were measured as total radioactivity in the remaining cell lysate by liquid scintillation counting, and total protein was quantified using a Pierce BCA Protein Assay Kit. The OATP1B-mediated uptake was calculated by dividing the disintegrations per minute (dpm) from each replicate by the amount of protein (mg) and subtracting the dpm/mg protein in the VC cell line. The OATP1B1-mediated uptake at each concentration of each antifungal drug was then compared with the OATP1B1-mediated uptake when only an equal volume of vehicle (water or DMSO) was added without the antifungal drug. The half-maximal inhibitory concentration (IC_50_) was calculated using a nonlinear fit comparing concentrations of each antifungal drug versus response; each concentration of each drug was tested in replicates of 6–9 across 2–3 independent experiments.

Competitive counterflow assays were performed with 8-acetoxy-trisulfopyrene, an activatable fluorogenic probe for OATP1B1, as described previously [20]. In brief, cells expressing a vector or OATP1B1 were seeded in 96-well opaque plates in phenol-red free DMEM media with 10% FBS and induced with doxycycline for 24 h. On the day of the experiment, the media was removed, and the cells were washed three times with warm PBS. The cells were then incubated for 20 min at 37 °C with 100 µL of 5 µM 8-acetoxypyrene-1,3,6-trisulfonate uptake buffer (ACE; 125 mM NaCl, 4.8 mM KCl, 1.2 mM CaCl_2_, 1.2 mM KH2PO_4_, 12 mM MgSO_4_, 25 mM 2-N-morpholino), ethanesulfonic acid, and 5.6 mM glucose, with the pH adjusted to 5.5). The dye uptake reaches equilibrium during this time, and stays stable for an additional 20 min. The fluorescence was measured at excitation and emission wavelengths of 460 nm and 510 nm, respectively, after 20 min of incubation to control for the number of cells.

For the counterflow phase of the experiment, the supernatant containing ACE alone was removed and 5 µM ACE with or without different concentrations of venetoclax, 0.5% paraformaldehyde or 10 µM cyclosporin A, a positive control substrate, added. The plates were incubated for 20 min at 37 °C. In the final step, the supernatant was removed, and the cells were washed three times with 200 µL ice-cold PBS to stop the reaction. PBS was then removed and 200 µL of 0.1 N NaOH was added to each well to normalize the pH. The fluorescence was measured as described above after another 20 min incubation at room temperature. The levels of cell dye were reported as a percent of the measurement from cells containing ACE alone.

### 2.6. In Vitro to In Vivo Extrapolation

To determine the clinical relevance of IC_50_ values, we utilized the basic model approaches outlined in the FDA’s 2020 guidance document “In Vitro Drug Interaction Studies—Cytochrome P450 Enzyme- and Transporter-Mediated Drug Interactions Guidance for Industry” [21], as has been done previously [7]. In brief, we utilized the equation C_max,ss_/IC_50_. To use this equation, we identified the mean total peak concentration at the steady-state of each potential inhibitor (C_max,ss_) from the published literature on the clinical pharmacokinetics of each agent (Supplementary Table S2). We then divided C_max,ss_ by the IC_50_ of the respective agents.

(1) $$\frac{C_{\mathrm{Max},\mathrm{ss}}}{{\mathrm{IC}}_{50}}$$

As indicated by the FDA guidelines, values of >0.1 indicate the potential for a clinically relevant interaction.

To further determine the potential clinical relevance of OATP1B1 inhibition by our panel of antifungal drugs and known OATP1B inhibitors, we calculated the R value based on the equation provided by this same guidance document:

(2) $$R=1+\left(\frac{f_{\mathrm{up}}\times \left(C_{\mathrm{Max}}+\frac{\mathrm{Dose}\;\times F_{a}\times F_{g}\times k_{a}}{Q_{h}}\right)}{{\mathrm{IC}}_{50}}\right)$$

To utilize this equation, we used the previously determined C_max,ss_ (Supplementary Table S2). Where necessary, the molar weight was used to convert the dose or C_max,ss_ to µM. We determined the unbound fraction in plasma (f_up_) for each drug from publicly available literature. Citations for C_max,ss_ and f_up_ can be found in Supplementary Table S2. As recommended by the FDA guidelines, the fraction absorbed (F_a_) and intestinal availability (F_g_) were set to 1, f_up_ was set to 1% when reported to be less than 1%, and the absorption constant (k_a_) was set to 0.1 min^−1^ (as a worst-case estimate). The hepatic blood flow rate (Q_h_) was set to 1.62 L/min [22]. An R value >1.1 indicates that a drug may have the potential to inhibit the transporter of interest in vivo.

### 2.7. Real-Time Polymerase Chain Reaction (qPCR)

Livers were harvested from female CYP3A(−/−), FVB wild-type, OATP1B2(−/−), and DBA wild-type mice between 8 and 12 weeks of age. Livers were snap frozen and stored at −80 °C until RNA isolation. RNA was isolated by utilizing an E.Z.N.A. Total RNA Kit I (Omega Bio-tek, Norcross, GA, USA), followed by cDNA synthesis with qScript XLT cDNA SuperMix (Quantabio, Beverly, MA, USA). Real-time polymerase chain reaction (qPCR) was performed using TaqMan probes for OATP1B2 (Mm00451513_m1), CYP3A11 (Mm00731567_m1), and GAPDH (Mm99999915_g1) (Thermo Fisher Scientific) and a QuantStudio 3 instrument (Thermo Fisher Scientific).

### 2.8. Statistical Analysis

All data represent the mean and standard error of the mean (SEM), unless stated otherwise. For pharmacokinetic studies comparing only two groups (i.e., CYP3A(−/−) versus wild-type), unpaired t-tests were performed comparing the dpm/mg protein, C_max_, or AUC_0-last_ of each treatment group. For studies comparing four groups, an unadjusted two-way ANOVA was performed with genotype (wild-type or CYP3A(−/−), wild-type or OATP1B2(−/−)) and treatment group (ketoconazole versus the respective vehicle) as between-subjects variables to determine the main effects and to compare the C_max_ and AUC of each treatment group. Statistical analyses were performed using GraphPad Prism 8.0 (GraphPad Software, San Diego, CA, USA). All statistical tests were two-tailed, and *P* < 0.05 was considered statistically significant.

## 3. Results

### 3.1. Contribution of CYP3A to the Interaction between Ketoconazole and Venetoclax

**Figure 1:**
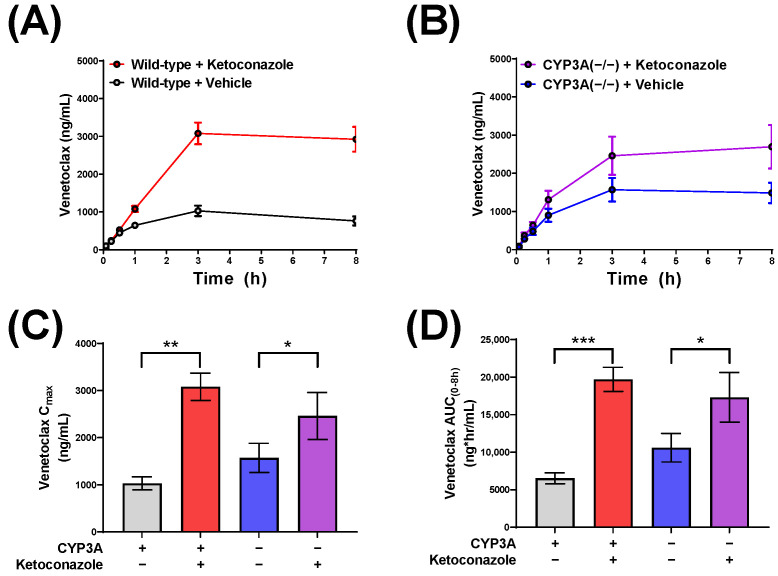
The ketoconazole-induced increases in venetoclax exposure are partially mediated by CYP3A. Plasma concentration curves of venetoclax in (**A**) female FVB wild-type or (**B**) CYP3A(−/−) mice administered venetoclax (10 mg/kg; PO) 30 min after ketoconazole (50 mg/kg; PO) or vehicle (PEG400; PO) (*n* = 5/group). Serial plasma samples were collected from each mouse and analyzed via LC-MS/MS. (**C**) Maximum concentration (C_max_) and (**D**) area under the concentration-time curve (AUC) using the last observed timepoint (AUC_0–8h_) calculated with non-compartmental analysis (NCA) using Phoenix WinNonlin 8.1; * *p* < 0.05, ** *p* < 0.01, *** *p* < 0.001. In a separate experiment, CYP3A(−/−) mice showed significantly greater venetoclax exposure when compared against wild-type FVB mice (Supplementary Figure S2; *p* = 0.01).

In our previous study, we established that mice could serve as a translationally relevant model organism to study the pharmacokinetics of venetoclax and demonstrated that ketoconazole increased the AUC of venetoclax in both male and female mice [13]. Based on the supposition that ketoconazole increases venetoclax exposure through CYP3A inhibition, we next evaluated the ability of human recombinant CYP3A4 to metabolize venetoclax. As expected, in the presence of CYP3A4, the amount of venetoclax in the reaction was reduced by 40% (*p* = 0.02). However, when incubated in the presence of ketoconazole, the amount of venetoclax in the reaction was nearly unchanged (*p* = 0.15) (Supplementary Figure S1). Based on these results, we determined the impact of CYP3A in vivo by assessing venetoclax pharmacokinetics in female mice lacking the eight murine CYP3A genes (CYP3A(−/−)). Compared to untreated wild-type mice, the AUC of venetoclax was increased by 1.8-fold in untreated CYP3A(−/−) mice (*p* = 0.01) (Supplementary Figure S2). To determine the contribution of CYP3A to the DDI between ketoconazole and venetoclax, we administered venetoclax to female wild-type and CYP3A(−/−) mice pre-treated with either ketoconazole or vehicle. Similar to our previous experiment, CYP3A(−/−) mice had a 1.6-fold increase in venetoclax exposure (*p* = 0.08). This experiment demonstrated that ketoconazole administration was associated with a 3-fold increase in the AUC of venetoclax in wild-type mice when compared with mice receiving only vehicle (*p* < 0.0001) (Figure 1). Surprisingly, the AUC of venetoclax was significantly increased by 1.6-fold in CYP3A(−/−) mice pre-treated with ketoconazole compared with vehicle (*p* = 0.04) (Figure 1B; Table 1). These data suggest that ketoconazole affects the systemic exposure to venetoclax through additional mechanisms independent of the effects on CYP3A.

### 3.2. Influence of OATP1B1 and OATP1B2 on Venetoclax Uptake and Exposure

**Figure 2:**
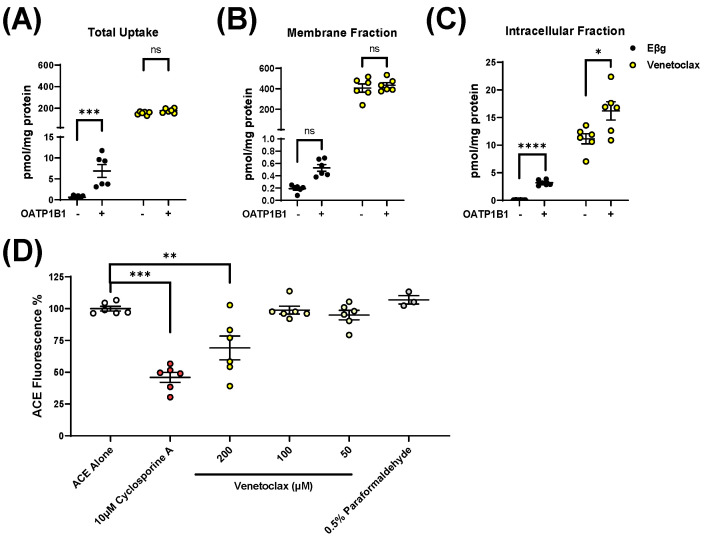
Venetoclax is an OATP1B1 substrate that extensively binds to the extracellular membrane. (**A**) In vitro uptake data utilizing 0.2 µM Eβg or venetoclax as substrates, measuring radioactivity of the total whole cell lysate (*n* = 6 across 2 biological replicates). (**B**,**C**) In vitro uptake data utilizing 0.2 µM Eβg or 1 µM venetoclax as substrates, measuring radioactivity of the either the (**B**) membrane fraction or (**C**) intracellular fraction of the total whole cell lysate after separation using a ProteoExtract^®^ Native Membrane Protein Extraction Kit (*n* = 6 across 2 biological replicates). (**D**) In vitro uptake data demonstrating that venetoclax is a competitive substrate of OATP1B1 (*n* = 6 across 2 biological replicates). A total of 10 µM Cyclosporine A was added as a control competitive inhibitor and 0.5% paraformaldehyde as a control for fixing the cells and inhibiting further movement of ACE across the membrane. * *p* < 0.05, ** *p* < 0.01, *** *p* < 0.001, **** *p* < 0.0001.

Since ketoconazole is known to potently inhibit various xenobiotic transporters, including OATP1B1 [7], and considering the potential transport of venetoclax by OATP1B-type transporters, we next sought to evaluate the contribution of OATP1B to the pharmacokinetics of venetoclax. First, we measured total radioactivity after incubating radioactive EβG, a probe substrate, or radioactive venetoclax with cells overexpressing OATP1B1 or the corresponding vector. Surprisingly, when incubated with venetoclax, both cells expressing OATP1B1 or the vector had >10-fold higher levels of detected radioactivity than the values observed for the probe substrate (Figure 2A). Given that venetoclax is >99% protein-bound in plasma [23], we hypothesized that venetoclax extensively binds to the extracellular membrane of cells in vitro, which confounds results when measuring total radioactivity. To test this hypothesis, we performed a similar uptake experiment and separated the intracellular fraction of cells from the membrane fraction. As expected, in the membrane fraction, the detected venetoclax-derived radioactivity was >500-fold greater than that measured for the probe substrate EβG. Applying a correction for the intracellular fraction, as much as >96% of the detectable venetoclax was associated with the membrane fraction (versus <15% of EβG) (Figure 2B). Based on an analysis of only the intracellular fraction, the overexpression of OATP1B1 was associated with significant increases in the uptake of both EβG (*p* < 0.001) and venetoclax (*p* < 0.05) (Figure 2C). Given the relative complexity of directly measuring the uptake of venetoclax, we also sought an indirect method of venetoclax quantification by performing competitive counterflow assays with 8-acetoxy-trisulfopyrene, a recently reported procedure that allows identification of OATP1B1 substrates. Whereas 0.5% paraformaldehyde, an OATP1B1 inhibitor, did not decrease fluorescence, both venetoclax (*p* < 0.01) and the positive control OATP1B1 substrate, cyclosporin A (*p* < 0.001) significantly decreased fluorescence (Figure 2D). These results suggest that venetoclax is a transported substrate of OATP1B1. Despite the high concentrations utilized in this experiment (200 µM), our membrane-binding experiment demonstrates that only <3% of venetoclax remains unbound, suggesting that the actual unbound concentration of venetoclax in these experiments was <10 µM.

**Figure 3:**
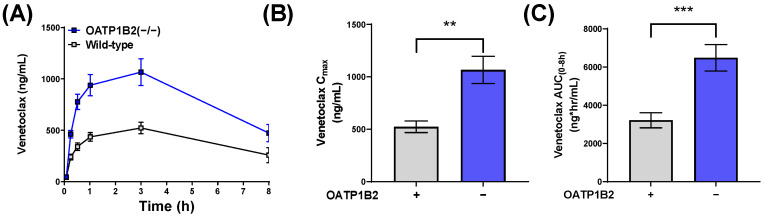
The genetic deletion of OATP1B2 increases venetoclax exposure. (**A**) Plasma concentration curves of venetoclax in female wild-type DBA or OATP1B2(−/−) mice administered venetoclax (10 mg/kg; PO) (*n* = 10/group). Serial plasma samples were collected from each mouse and analyzed via LC-MS/MS. (**B**) Maximum concentration (C_max_) and (**C**) area under the concentration-time curve (AUC) using the last observed timepoint (AUC_0–8h_) calculated with non-compartmental analysis (NCA) using Phoenix WinNonlin 8.1., ** *p* < 0.01, *** *p* < 0.001.

Based on the identified interaction of venetoclax with OATP1B-type transport, we next examined the pharmacokinetics of venetoclax in both male and female mice lacking OATP1B2 (OATP1B2(−/−)), the single murine orthologue of OATP1B1 and OATP1B3. Compared with the corresponding wild-type mice, the venetoclax AUC was increased by 2-fold in female OATP1B2(−/−) mice (*p* < 0.001) (Figure 3), and a similar increase was observed in male OATP1B2(−/−) mice (Supplementary Figure S3). To confirm that the pharmacokinetic observations in the OATP1B2(−/−) and CYP3A(−/−) mice were due to genetic deficiency of the respective transporter and DMEs, unaffected by potential compensatory upregulation of other genes and pathways of relevance to venetoclax pharmacokinetics, we determined the expression of the main CYP3A isoform, CYP3A11, in the livers of OATP1B2(−/−) and matched wild-type mice utilizing qPCR (Supplementary Figure S4A). Consistent with previous observations [17], we did not detect statistically significant differences in hepatic expression of CYP3A11 in OATP1B2(−/−) and wild-type mice. Similarly, we determined that the expression of the OATP1B2 gene was similar in the livers of CYP3A(−/−) mice and age-and sex-matched wild-type mice (Supplementary Figure S4B).

### 3.3. Impact of Clinically-Relevant Antifungal Drugs on OATP1B1 and OATP1B2 Function

Based on the murine pharmacokinetic data obtained with venetoclax, we hypothesized that antifungal agents other than ketoconazole that are used clinically in combination with venetoclax may have a similar propensity to affect OATP1B-type transporter function. To establish a panel of clinically-relevant antifungal agents, we reviewed clinical guidelines that describe antifungal prophylaxis for patients with cancer-related immunosuppression [24]. Based on these data, we created a panel of six azole antifungals (ketoconazole, itraconazole, fluconazole, posaconazole, isavuconazole, and voriconazole) and three echinocandins (micafungin, caspofungin, anidulafungin) for evaluation as OATP1B1 inhibitors. We also included rifampin, ritonavir, and cobicistat as positive control OATP1B1 inhibitors [7,25,26].

We performed radioactive uptake assays to determine the OATP1B1 inhibitory potential of our antifungal drug panel. Inhibition curves over a range of drug concentrations are shown in Supplementary Figure S5 with IC_50_ values summarized in Table 2. As expected, ketoconazole, rifampin, ritonavir, and cobicistat each inhibited OATP1B1 function with an IC_50_ < 3 µM, whereas itraconazole did not potently inhibit OATP1B1 function with an IC_50_ >20 µM. In line with previous observations [7], we found that the major itraconazole metabolites, hydroxy-itraconazole and keto-itraconazole, were more potent inhibitors of OATP1B1 than the parent drug. Consistent with our original hypothesis suggesting differential OATP1B1 inhibition by antifungal drugs, we found that isavuconazole, posaconazole, and micafungin inhibited OATP1B1 function with an IC_50_ < 3 µM, whereas fluconazole and voriconazole did not potently inhibit OATP1B1 at observed IC_50_ values > 50 µM.

To determine the clinical relevance of these IC_50_ values, we utilized an equation outlined by the FDA’s 2020 guidelines (Equation (1)) to derive values for C_max,ss_/IC_50_ (Table 2). While the inhibitory properties of most of the drugs included in our panel were considered to be of potential clinical relevance (value > 0.1), substantial variability was observed. For example, micafungin, isavuconazole, ketoconazole, and posaconazole each had values >1, suggesting greater DDI potential compared to other antifungals. To further evaluate clinical relevance, we determined the R value (Equation (2)) for each drug using the data provided in Supplementary Table S2; the chemical structure of each inhibitor is provided in Supplementary Figure S6. Voriconazole, posaconazole, isavuconazole, and micafungin each had a C_max,ss_/IC_50_ > 0.1 and were considered to be associated with a potentially clinically-relevant interaction with OATP1B1 (R values > 1.1). Supporting these data, as expected, our control inhibitors (rifampin, ritonavir, ketoconazole, and cobicistat) each had a C_max,ss_/IC_50_ > 0.1 and an R value > 1.1.

To create in vivo studies characterizing the relative contribution of OATP1B-type transporters to the DDI potential of antifungals, we tested the ability of the identified OATP1B1 inhibitors to also inhibit the function of OATP1B2. Inhibition curves over a range of drug concentrations are shown in Supplementary Figure S7, and IC_50_ values are presented in Table 2. As expected, based on their OATP1B1 inhibition profile, ketoconazole, isavuconazole, cobicistat, rifampin, and micafungin each inhibited OATP1B2 function with IC_50_ values of <5 µM. However, posaconazole did not inhibit OATP1B2 function (IC_50_ > 80 µM), suggesting a potential species-dependent inhibition profile of this drug toward OATP1B-type transporters. As expected, based on the observed weak inhibitory properties toward OATP1B1, voriconazole and fluconazole did not potently inhibit OATP1B2 function (IC_50_ > 100 µM).

### 3.4. Impact of Micafungin on Venetoclax Exposure

**Figure 4:**
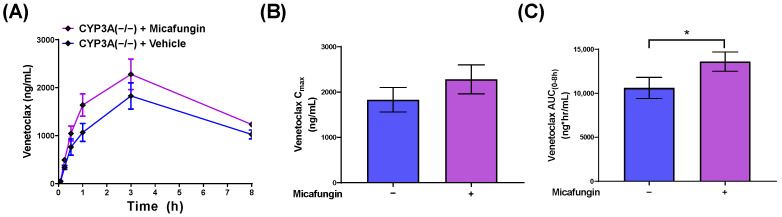
Micafungin-induced increases in venetoclax exposure are not mediated by CYP3A. (**A**) Plasma concentration curves of venetoclax in female CYP3A(−/−) mice administered venetoclax (10 mg/kg; PO) 30 min after micafungin (100 mg/kg; IV) or vehicle (PBS; IV) (*n* = 5/group). Serial plasma samples were collected from each mouse and analyzed via LC-MS/MS. (**B**) Maximum concentration (C_max_) and (**C**) area under the concentration-time curve (AUC) using the last observed timepoint (AUC_0–8h_) calculated with non-compartmental analysis (NCA) using Phoenix WinNonlin 8.1. * *p* < 0.05.

**Figure 5:**
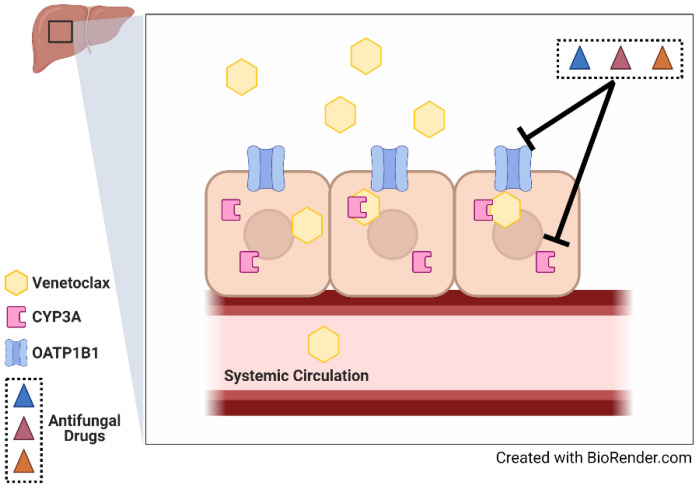
Summary figure. Both CYP3A and OATP1B1 contribute to the pharmacokinetics of venetoclax and are inhibited by antifungal drugs. Solid lines represent knowledge gaps clarified by our experiments.

For in vivo validation studies, we selected micafungin, an echinocandin that does not significantly inhibit CYP3A-mediated metabolism, and that was identified as an OATP1B-type transporter inhibitor. Consistent with an OATP1B-dependent DDI, CYP3A(−/−) mice pre-treated with micafungin had a statistically significant 1.3-fold increase in the AUC of venetoclax compared to CYP3A(−/−) mice that were pre-treated with saline (*p* = 0.04) (Figure 4). Based on our collective findings, we suggest that both CYP3A and OATP1B1 contribute to the pharmacokinetics of venetoclax and could be impacted by concomitant antifungal usage; these findings are summarized in Figure 5.

## 4. Discussion

The current study adds to a growing body of literature suggesting that antifungal drugs have a propensity to cause DDIs through CYP3A-independent mechanisms, highlighting a previously unrecognized type of DDI of relevance to the antileukemic drug, venetoclax. In particular, we found that pretreatment with certain antifungals, such as ketoconazole, is associated with statistically significant increases in the systemic exposure to venetoclax in both wild-type mice and CYP3A(−/−) mice, suggesting the existence of an additional mechanism that contributes to the observed DDI. Employing an array of in vitro and in vivo transport models, this mechanism was identified as OATP1B-mediated transport, a conclusion that is consistent with the notion that several prototypical CYP3A inhibitors, including ketoconazole, are well-established inhibitors of this class of transporters [7].

Our data also unambiguously validated the thesis that the elimination pathway of venetoclax is dependent, at least in part, on CYP3A-mediated metabolism, a conclusion that is consistent with a previously reported clinical DDI study of venetoclax given in combination with ketoconazole [5]. In contrast, previously available evidence in support of a contribution of OATP1B-type transport to the pharmacokinetics of venetoclax has been conflicting. Venetoclax can be classified in the Biopharmaceutics Classification System (BCS) as a category IV drug characterized by low aqueous solubility and poor membrane permeability properties [38], yet in tissue distribution studies, the highest concentrations of venetoclax are found in the liver [39]. This suggests the involvement of xenobiotic uptake transporters such as OATP1B1 in the hepatic accumulation, and subsequent elimination, of venetoclax [21]. Although the venetoclax prescribing information states that venetoclax is likely not a transported substrate of OATP1B1, other regulatory documents suggest that venetoclax can inhibit the function of OATP1B1 in vitro, and that its clearance in vivo is reduced by 60% in mice with a deficiency of all OATP1A and OATP1B type transporters [23]. Our present pharmacokinetic data obtained in OATP1B2(−/−) mice are consistent with this prior knowledge, and also suggest that the previously observed lack of transport of venetoclax by OATP1B1 in cell-based models is possibly caused by methodological differences in the applied approaches. In particular, our data suggest that venetoclax exhibits extensive, non-specific membrane-binding properties that complicate the analysis and interpretation of results from in vitro uptake experiments. Indeed, the major fraction of venetoclax added to cells binds non-specifically to the outer plasma membrane of cells, providing a plausible explanation for past failures to identify uptake transporters of venetoclax from in vitro studies that may have in vivo significance. Previous studies have reported phenomena similar to that observed here with venetoclax; for example, even within minutes, up to 75% of doxorubicin presented to cells is associated with the membrane fraction [40], and this non-specific membrane binding results in artificially high “uptake” values in vitro and ex vivo in the absence of transporter overexpression [19]. As such, this phenomenon provides an explanation for the thesis that the genetic deficiency of certain hepatic transporters can cause altered elimination properties for substrates in vivo to an extent that cannot be predicted by commonly used in vitro uptake studies. Furthermore, it suggests that the extensive non-specific binding of agents such as venetoclax and other targeted drugs, such as members of the class of kinase inhibitors [41], should be taken into consideration and corrected for in the context of cell-based transport studies.

Based on these collective data and the expanding appreciation for the contribution of OATP1B1 to the elimination of targeted anticancer drugs [8], we focused on characterizing the ability of newer, more clinically-relevant antifungal drugs to inhibit OATP1B1. These studies resulted in the identification of posaconazole and isavuconazole as inhibitors of OATP1B1 that can affect transport function at clinically-achievable concentrations, confirming the inhibitory properties of various established OATP1B1 modulators, including ketoconazole, ritonavir [7], cobicistat [26], and rifampin [25]. For posaconazole, these data validate previous computational modeling that predicted OATP1B1 inhibition by this drug [42]. For isavuconazole, previous data has suggested only weak inhibition of OATP1B1 (IC_50_ > 10 µM) with no effect on the pharmacokinetics of repaglinide (a substrate of OATP1B1 and CYP2C8) [43]. It should be pointed out, however, that isavuconazole is associated with increases in the systemic exposure of atorvastatin, a substrate of OATP1B1 and CYP3A [44], although the relative contributions of OATP1B1 and CYP3A to the isavuconazole–atorvastatin DDI remain uncertain. Similarly, posaconazole increased simvastatin, a substrate of OATP1B1 and CYP3A, exposure by nearly 11-fold [45], and our present findings are consistent with the possibility that the inhibition of the OATP1B-mediated transport of simvastatin contributed to this DDI. While our data supports the thesis that the inhibition of OATP1B1 by these azole antifungals is clinically relevant and may contribute to their DDI potential, the ability of these drugs to also inhibit CYP3A-mediated metabolism complicates the interpretation of these clinical data. Additional in vivo studies are required to clarify this uncertainty, for example, by determining the impact of these drugs on biomarkers for OATP1B function and by utilizing models deficient for OATP1B or CYP3A, as we have employed in the current study.

In addition to the observations made with azole antifungals, we observed that micafungin, an echinocandin that lacks substantial CYP3A inhibition, also has an intrinsic ability to modulate OATP1B1 function at clinically relevant concentrations. Although prior studies indicated that certain echinocandins can inhibit efflux transporters [46], relatively little was known about their DDI potential through hepatic uptake transporters like OATP1B1. Computational modeling identified all three of these echinocandins as potential OATP1B1 inhibitors [42], and for micafungin, we confirmed this prediction in the present study. In this context, it is noteworthy that we recently determined that the pharmacokinetic profile of sorafenib and its glucuronide metabolite in patients with cancer is affected by concurrent usage of micafungin, posaconazole, and voriconazole by a mechanism that involves modulation of the OATP1B1 function [47]. This is particularly interesting because micafungin was selected as a preferred antifungal in subjects receiving sorafenib-based treatment in order to avoid an anticipated CYP3A-mediated DDI between sorafenib and azole antifungals. Consistent with the ability of micafungin to cause a clinically relevant degree of OATP1B1 inhibition in humans, micafungin was previously shown to increase exposure to sirolimus and cyclosporine [48], which are known OATP1B1 substrates [20]. Although in our present study, micafungin pretreatment was associated with a relatively modest increase in the systemic exposure to venetoclax, it should be pointed out that the observed DDI was of the same order of magnitude as that observed for ketoconazole.

While our findings suggest that both CYP3A and OATP1B contribute independently to the elimination of venetoclax, it remains unclear if additional mechanisms could possibly contribute to DDIs with venetoclax. In this context, it is worth noting that the DDI between venetoclax and ketoconazole is underpredicted in mice (~3-fold) [13] versus humans (~6-fold) [5]. This disparity suggests the existence of potential species-dependent differences in mechanisms regulating the elimination of venetoclax, a thesis that is consistent with the previous observation that venetoclax undergoes more extensive biotransformation to the metabolite M27 in humans (~40% of venetoclax levels) than in mice (<1% of venetoclax levels) [39]. Despite these differences, a single dose of rifampin, a prototypical inhibitor of OATP1B1, was previously found to be associated with a 2-fold increase in systemic exposure to venetoclax in humans [49]. This observation is consistent with our present findings in mice with a genetic deficiency of OATP1B2 and in mice lacking CYP3A receiving concurrent treatment with OATP1B-inhibitory antifungal drugs. Although contributions of ATP-binding cassette transporters such as ABCB1 (P-gp; MDR1) to the observed DDIs with venetoclax cannot be definitively excluded [39], clinical studies have indicated that ABCB1 inhibitors such as azithromycin do not influence the plasma levels of venetoclax [50].

It is tempting to speculate that, beyond the recorded antifungals, CYP3A and OATP1B might also contribute to other DDIs with venetoclax. Further understanding the significance of these mechanisms is particularly important, as venetoclax is frequently used clinically as the therapeutic backbone of combination regimens [51]. For example, there is considerable interest in combining venetoclax with FLT3 inhibitors to improve outcomes in AML, and these drugs may impair the function of OATP1B1 and cause unanticipated DDIs that can remain unnoticed, compromising the safety of such combinatorial modalities. For example, in vivo studies have shown that OATP1B-type transporters contribute to the pharmacokinetic profile of currently approved FLT3 inhibitors such as sorafenib [12] and gilteritinib [52]. It is thus recommended that future studies seek to elucidate and characterize the DDI liability for regimens involving venetoclax by using properly designed strategies and predictive model systems [10].

Overall, our findings demonstrate that CYP3A and OATP1B1 contribute independently to the elimination of venetoclax and to DDIs between venetoclax and a number of widely used, clinically relevant antifungal drugs. These findings provided the foundation for currently ongoing, prospective clinical trials aimed at optimizing drug doses by refining the concurrent usage of venetoclax with antifungal regimens in patients.

## Figures and Tables

**Table 1:** Venetoclax pharmacokinetic parameters.

Mouse Genotype	Sex	N	Co-Treatment (Dose mg/kg)	Venetoclax C_max_ (ng/mL)	Venetoclax AUC_(0–8h)_ (ng*h/mL)	Venetoclax AUC Fold Increase
Wild-type FVB	F	5	PEG400	1030 (140)	6530 (720)	
Wild-type FVB	F	5	Ketoconazole (50)	3080 (290) **	19,700 (1600) ***	3.0 (versus vehicle-treated wild-type FVB)
CYP3A(−/−)	F	5	PEG 400	1570 (310)	10,600 (1900)	1.6 (versus vehicle-treated wild-type FVB)
CYP3A(−/−)	F	5	Ketoconazole (50)	2700 (570) *	17,300 (3300) **	1.6 (versus vehicle-treated wild-type CYP3A(−/−))
Wild-type DBA	F	10	None	523 (55)	3211 (395)	
OATP1B2(−/−)	F	10	None	1066 (130) **	6481 (695) ***	2.0 (versus wild-type DBA)
CYP3A(−/−)	F	5	None	1830 (270)	10,600 (1200)	
CYP3A(−/−)	F	5	Micafungin (100)	2280 (320)	13,600 (1100) *	1.3 (versus vehicle-treated wild-type CYP3A(−/−))

Values are the mean with standard error in parenthesis. Abbreviations: C_max_—maximum plasma concentration; AUC_0–8h_—area under the concentration-time curve (AUC) from time zero to the 8 h (the last observed timepoint); statistics compare groups as explicitly stated * *p* < 0.05, ** *p* < 0.01, *** *p* < 0.001.

**Table 2:** The inhibition of OATP1B1 and OATP1B2 by antifungal drugs and potential clinical relevance.

Inhibitor	OATP1B1 IC_50_ (µM)	95% CI	OATP1B2 IC_50_ (µM)	95% CI	C_max,ss_ (µM) ^a^	Citation	C_max,ss_/IC_50_ (>0.1 Considered Clinically Relevant)
Ketoconazole	1.5	(1.2, 1.7)	2.2	(1.4, 3.4)	1.88	[7]	1.25
Cobicistat	0.31	(0.24, 0.40)	0.7	(0.57, 0.83)	1.29	[27]	1.84
Ritonavir	0.34	(0.29, 0.40)	ND	ND	1.94	[28]	2.71
Rifampin	1.2	(0.9, 2.2)	2.1	(1.5, 3)	29	[29]	24.2
Itraconazole	37	(31, 45)	>50	ND	1.15	[30]	0.03
Hydroxy-ITZ	3.5	(3, 3.9)	>2	ND	0.6	[30]	0.17
Keto-ITZ	8.3	(6.9, 10)	ND	ND	0.02	[30]	0.002
Posaconazole	1.9	(1.6, 2.2)	>20	ND	2.75	[31]	1.45
Voriconazole	80	(62, 104)	>200	ND	10.2	[32]	0.13
Fluconazole	4100	(3200, 5300)	>5000	ND	61.7	[33]	0.06
Isavuconazole	2.5	(2.1, 3.0)	2.5	(1.1, 5.8)	8.23	[34]	3.29
Micafungin	2.1	(1.8, 2.4)	4.9	(4.2, 5.7)	17.3	[35]	8.24
Caspofungin	9.2	(8.0, 11)	>100	ND	7.99	[36]	0.87
Anidulafungin	42	(31, 57)	ND	ND	6.14	[37]	0.15

^a^ Maximum plasma concentration values are representative of clinical regimens used for antifungal prophylaxis. Further information regarding the selection of these values is located in Supplementary Table S2. Abbreviations: C_max,ss_—maximum plasma concentration at steady-state; IC_50_—half-maximal inhibitory concentration; ND—not determined.

## Data Availability

The data presented in this study are available on request from the corresponding author.

## Supplementary Materials

The following supporting information can be downloaded at: https://www.mdpi.com/article/10.3390/pharmaceutics14040694/s1, Figure S1: Venetoclax is metabolized by human CYP3A4 in vitro; Figure S2: Venetoclax exposure is partially mediated by CYP3A; Figure S3: Genetic deletion of OATP1B2 increases venetoclax exposure in male mice; Figure S4: Expression of CYP3A11 and OATP1B2 in OATP1B2(−/−) and CYP3A(−/−) mice; Figure S5: Inhibition of OATP1B1 function by indicated antifungal drugs in vitro; Figure S6: Chemical structures of venetoclax and inhibitors; Figure S7: Inhibition of OATP1B2 function by indicated antifungal drugs in vitro;. Table S1: Clinical pharmacokinetics of antifungal agents; Table S2: Venetoclax pharmacokinetic parameters.
