# Supplementary material for: Interaction of Antifungal Drugs with CYP3A- and OATP1B-Mediated Venetoclax Elimination
Source: Pharmaceutics. 2022 Mar 23;14(4):694. doi: 10.3390/pharmaceutics14040694 (PMC9025810; doi:10.3390/pharmaceutics14040694)
Supplement: Supplementary file 1 [file pharmaceutics-14-00694-s001.zip › pharmaceutics-1568758-supplementary.pdf]

pharmaceutics

MDPI

Supplementary Materials: Interaction of Antifungal Drugs with CYP3A- and OATP1B-Mediated Venetoclax Elimination

Eric D. Eisenmann, Dominique A. Garrison, Zahra Talebi, Yan Jin, Josie A. Silvaroli, Jin Gyu Kim, Alex Sparreboom, Michael R. Savona, Alice S. Mims and Sharyn D. Baker

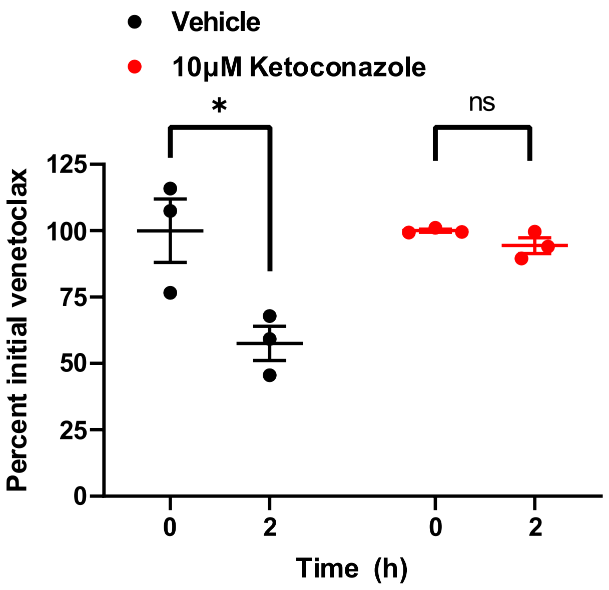

Figure S1. Venetoclax is metabolized by human CYP3A4 in vitro. 10 μM venetoclax was incubated with 40 nM human CYP3A4 supersomes and10 μM ketoconazole or vehicle (DMSO) for 0 h or 2 h. Venetoclax concentrations (ng/mL) were normalized to concentrations of venetoclax at 0 h. * $p <$ 0.05.

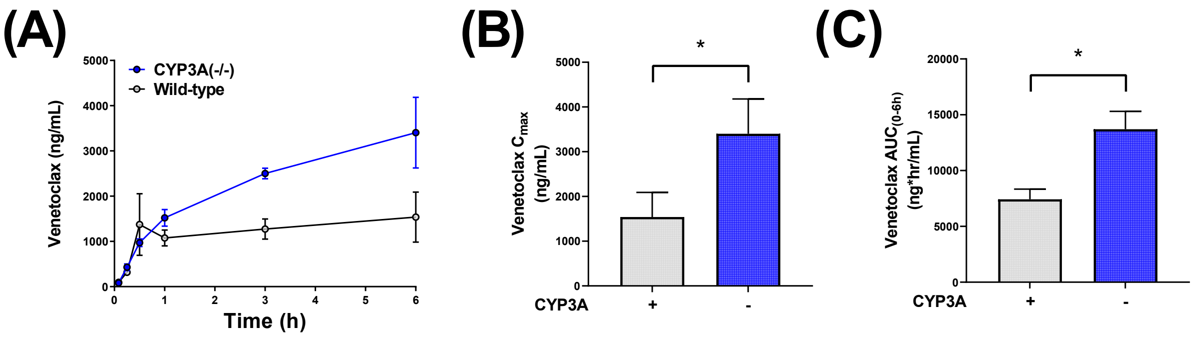

Figure S2. Venetoclax exposure is partially mediated by CYP3A. **(A)** Plasma concentration curves of venetoclax in female FVB wild-type or CYP3A(-/-) mice administered venetoclax (10 mg/kg; PO). Serial plasma samples were collected from each mouse and analyzed via LC-MS/MS. **(B)** Maximum concentration (C_{max}) and **(C)** area under the concentration-time curve (AUC) using the last observed timepoint (AUC_{0-6h}) calculated with non-compartmental analysis (NCA) using Phoenix WinNonlin 8.1. *$p < 0.05$.

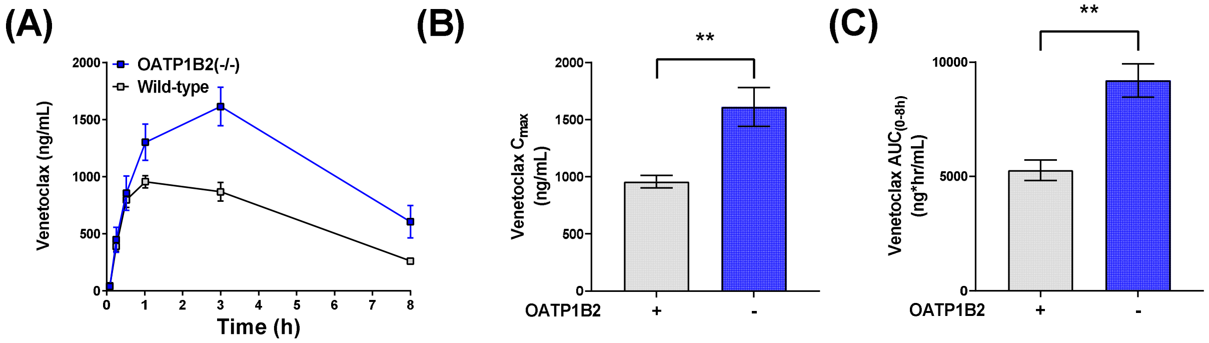

Figure S3. Genetic deletion of OATP1B2 increases venetoclax exposure in male mice. **(A)** Plasma concentration curves of venetoclax in male wild-type DBA or OATP1B2(-/-) mice administered venetoclax (10mg/kg; PO) (n=10/group). Serial plasma samples were collected from each mouse and analyzed via LC-MS/MS. **(B)** Maximum concentration (C_{max}) and **(C)** area under the concentration-time curve (AUC) using the last observed timepoint (AUC_{0-8h}) calculated with non-compartmental analysis (NCA) using Phoenix WinNonlin 8.1. ** $p < 0.01$.

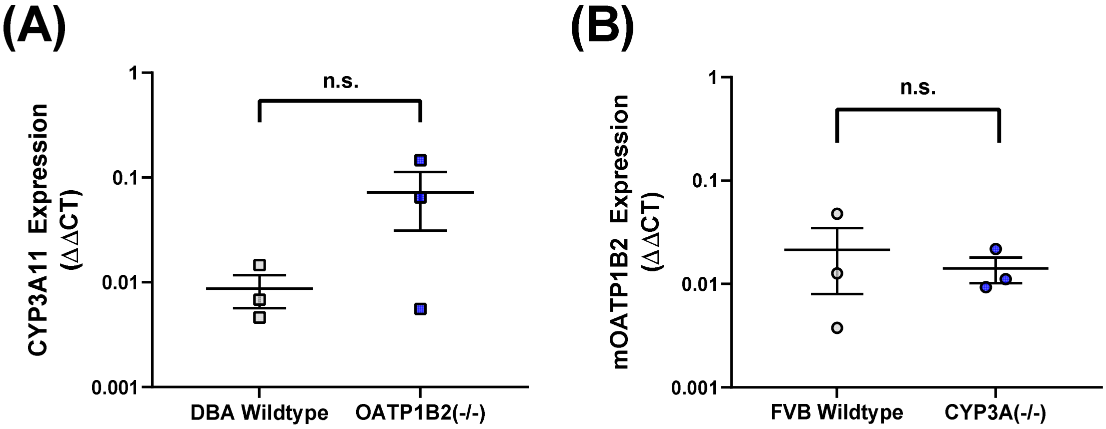

Figure S4. Expression of CYP3A11 and OATP1B2 in OATP1B2(-/-) and CYP3A(-/-) mice. Livers were harvested from female CYP3A(-/-), FVB wild-type, Oatp1b2(-/-), and DBA wild-type mice between 8 and 12 weeks of age (n = 3/group). mRNA expression was assessed by real-time qPCR.

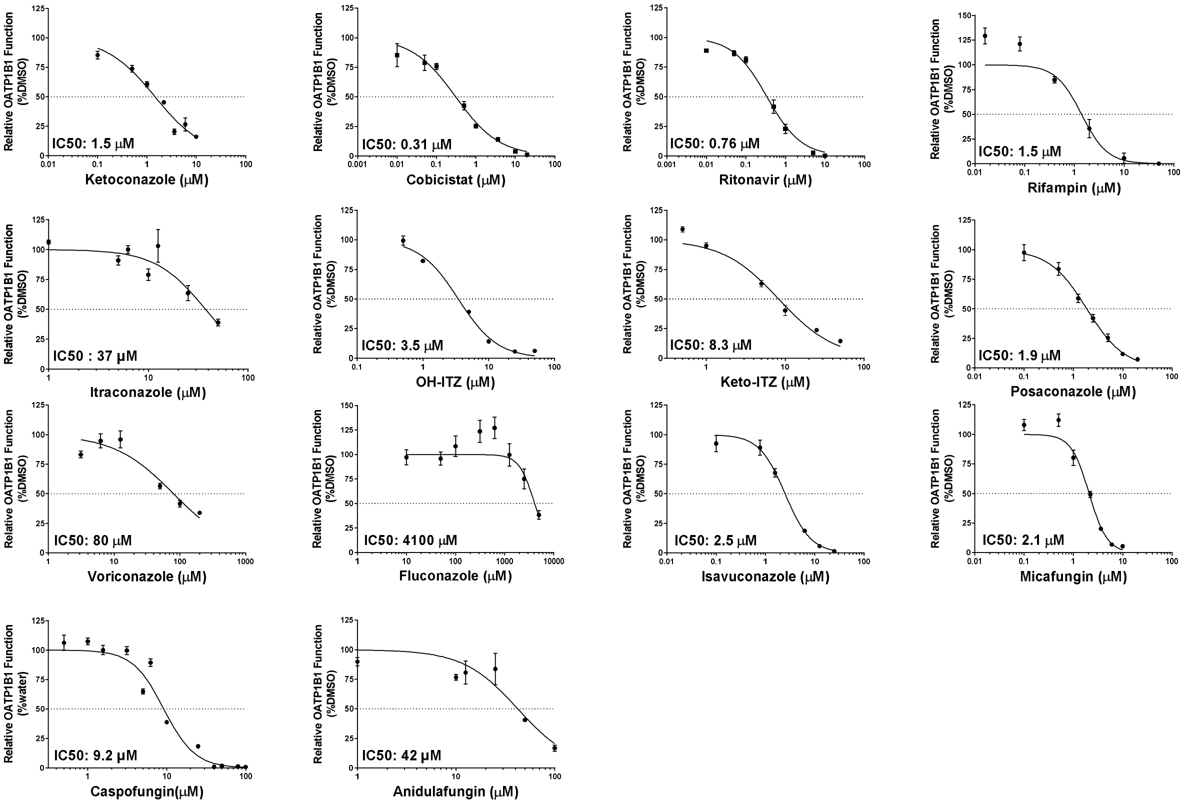

Figure S5. Inhibition of OATP1B1 function by indicated antifungal drugs in vitro. HEK293 cells expressing OATP1B1 or VC were pre-incubated with indicated antifungal drugs at the indicated concentrations for 15 minutes before incubation with the indicated antifungal and [^{3}H]Estradiol-17β-D-glucuronide for 15 minutes. Data represent uptake of OATP1B1-expressing cells at each concentration compared against vehicle after subtracting uptake by VC cells (mean ± SEM). Each concentration consists of 6–9 technical replicates across 2–3 biological replicates, except for OH-ITZ and Keto-ITZ which were only completed once due to limited reagent.

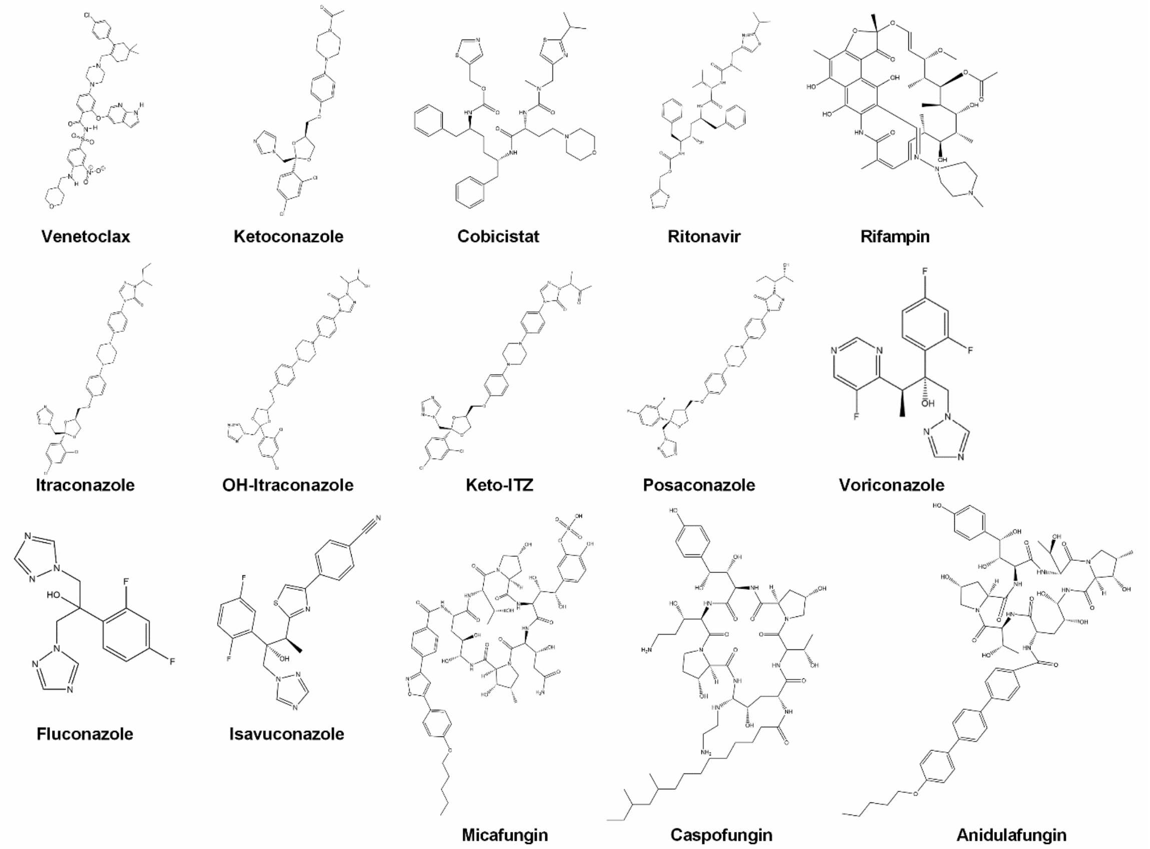

Figure S6. Chemical structures of venetoclax and inhibitors.

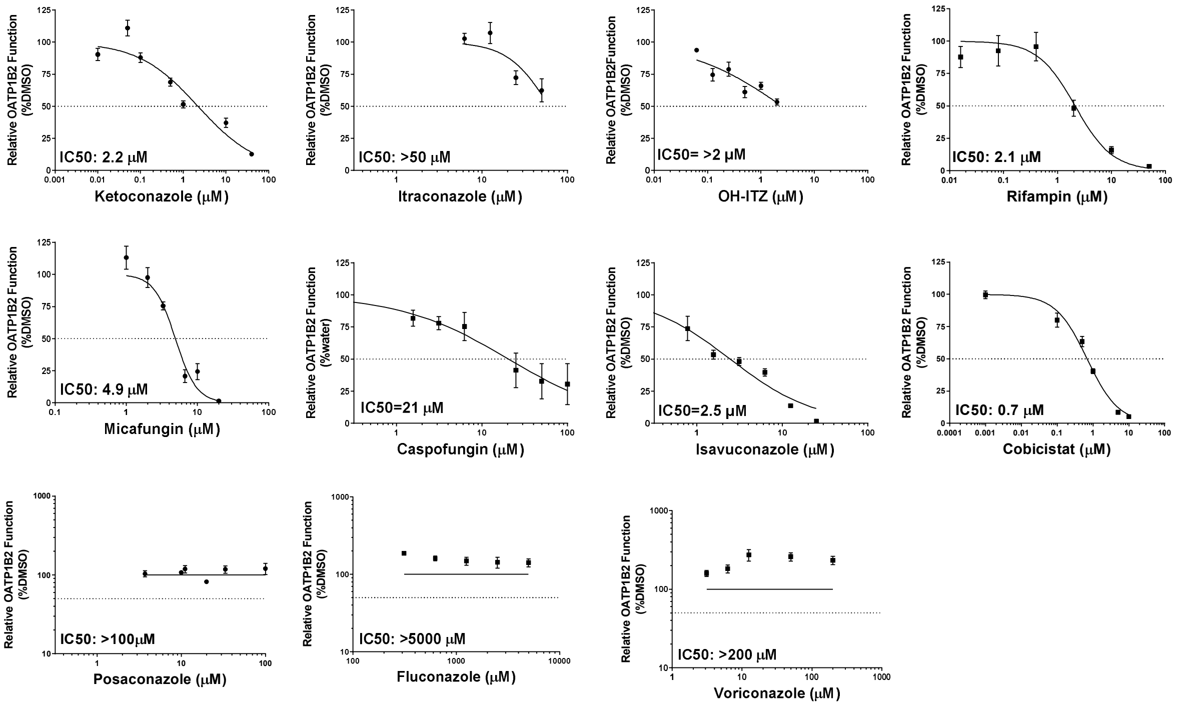

Figure S7. Inhibition of OATP1B2 function by indicated antifungal drugs in vitro. HEK293 cells expressing OATP1B2 or VC were pre-incubated with indicated antifungal drugs at the indicated concentrations for 15 minutes before incubation with the indicated antifungal and [^{3}H]Estradiol-

17β-D-glucuronide for 15 minutes. Data represent uptake of OATP1B2-expressing cells at each concentration compared against vehicle after subtracting uptake by VC cells (mean ± SEM). Each concentration consists of 6-9 technical replicates across 2-3 biological replicates, except for OH-ITZ which was only completed once due to limited reagent.

Table S1. Venetoclax pharmacokinetic parameters.

Mouse genotype	N	Sex	Co-treatment (dose mg/kg)	Venetoclax C_{max} (ng/mL)	Venetoclax $AUC_{(0-last)}$ (ng*h/mL)	Venetoclax AUC Fold increase
Wild-type FVB	5	F	None	1540 (550)	7430 (910)	
CYP3A(-/-)	3	F	None	3400 (780)*	13700 (1600)**	1.8
Wild-type DBA	5	M	None	958 (54)	5270 (450)	
OATP1B2(-/-)	5	M	None	1610 (170)**	9200 (730)***	1.7

Values are the mean with standard error in parenthesis. Abbreviations: C_{max}, maximum plasma concentration; $AUC_{0-last,}$ area under the concentration-time curve (AUC) from time zero to the last observed timepoint; * $p < 0.05$, ** $p < 0.01$, *** $p < 0.001$.

Table S2. Clinical pharmacokinetics of antifungal agents.

Inhibitor	Name Brand	Initial FDA Approval (year) [a]	Antifungal Prophylaxis Regimen	Citation (PMID)	Clinical PK Study Regimen	$C_{max,ss}$ (μM)	Citation (PMID)	Fraction Unbound [b] (fup) (%)	Citation (PMID)
Ketoconazole	Nizoral	1981	200mg-400mg QD [c]	25229352	200mg QD	1.88	26668209	3.2	11744613
Ritonavir	Norvir	1996	ND	ND	100mg BID	1.9	15105105	1	25989229
Cobicistat	Tybost	2012	ND	ND	150mg QD	1.29	26319088	2.71	24550332
Rifampin	Rifadin	1974	ND	ND	600mg QD (IV)	29	27526979	11	25801554
Itraconazole	Sporanox	1997	200mg BID for 2 days	12729424	100mg QD	1.15	17495874	3.6	17495874
Hydroxy-ITZ	ND	ND	ND	ND	ND	0.608	17495874	0.44	17495874
Keto-ITZ	ND	ND	ND	ND	ND	0.023	17495874	5.3	17495874
Posaconazole	Noxafil	2006	300mg QD	26612870	300mg QD	2.75	26612870	<2	32178468
Voriconazole	Vfend	2002	200mg BID	19747629	200mg BID	10.2	23766489	33-49	32178468
Fluconazole	Diflucan	1990	400mg QD	16339606	400mg QD	61.7	2196167	88-89	32178468
Isavuconazole	Cresemba	2015	200mg QD	32236406	200mg QD	8.23	25624327	<1	32178468
Micafungin	Mycamine	2005	100mg QD	29746955	100mg QD	17.3	28791666	<1	32178468
Caspofungin	Cancidas	2001	50mg QD	16377679	50mg QD	7.99	11850256	5	32178468
Anidulafungin	Eraxis	2006	200mg once, then 100mg QD	25376267	200mg once, then 100mg QD	6.14	21486730	1	32178468

[a] Based on the first available formulation. [b] f_{up} was calculated by subtracting the protein binding from 100%. [c] Ketoconazole is generally not used clinically for antifungal prophylaxis. Abbreviations: $C_{max,ss}$, maximum plasma concentration at steady-state; ND, not determined.
